# Racial/ethnic differences in experimental pain sensitivity and associated factors – Cardiovascular responsiveness and psychological status

**DOI:** 10.1371/journal.pone.0215534

**Published:** 2019-04-18

**Authors:** Hee Jun Kim, Joel D. Greenspan, Richard Ohrbach, Roger B. Fillingim, William Maixner, Cynthia L. Renn, Meg Johantgen, Shijun Zhu, Susan G. Dorsey

**Affiliations:** 1 Department of Nursing, Towson University, Towson, Maryland, United States of America; 2 Department of Neural and Pain Sciences, and Brotman Facial Pain Clinic, University of Maryland, Baltimore, Maryland, United States of America; 3 Department of Oral Diagnostic Sciences, University at Buffalo, Buffalo, New York, United States of America; 4 Department of Community Dentistry & Behavioral Science, University of Florida, Gainesville, Florida, United States of America; 5 Center for Translational Pain Medicine, Duke University, Durham, North Carolina, United States of America; 6 Department of Pain and Translational Symptom Science, University of Maryland School of Nursing, Baltimore, Maryland, United States of America; 7 Organizational Systems and Adult Health Department, University of Maryland School of Nursing, Baltimore, Maryland, United States of America; Heidelberg University, GERMANY

## Abstract

This study evaluated the contributions of psychological status and cardiovascular responsiveness to racial/ethnic differences in experimental pain sensitivity. The baseline measures of 3,159 healthy individuals—non-Hispanic white (NHW): 1,637, African-American (AA): 1,012, Asian: 299, and Hispanic: 211—from the OPPERA prospective cohort study were used. Cardiovascular responsiveness measures and psychological status were included in structural equation modeling based mediation analyses. Pain catastrophizing was a significant mediator for the associations between race/ethnicity and heat pain tolerance, heat pain ratings, heat pain aftersensations, mechanical cutaneous pain ratings and aftersensations, and mechanical cutaneous pain temporal summation for both Asians and AAs compared to NHWs. HR/MAP index showed a significant inconsistent (mitigating) mediating effect on the association between race/ethnicity (AAs vs. NHWs) and heat pain tolerance. Similarly, coping inconsistently mediated the association between race/ethnicity and mechanical cutaneous pain temporal summation in both AAs and Asians, compared to NHWs. The factor encompassing depression, anxiety, and stress was a significant mediator for the associations between race/ethnicity (Asians vs. NHWs) and heat pain aftersensations. Thus, while pain catastrophizing mediated racial/ethnic differences in many of the QST measures, the psychological and cardiovascular mediators were distinctly restrictive, signifying multiple independent mechanisms in racial/ethnic differences in pain.

## Introduction

Pain is a major health problem in the US, with an estimated 120 million (55.7%) adults reporting some level of pain in the previous three months, including chronic pain [1]. Racial/ethnic disparities related to pain in the US have been studied widely, with racial/ethnic minorities reporting greater severity of chronic pain than non-Hispanic white (NHWs) [2–4]. The problem is compounded by lower quality of care provided to patients who are racial/ethnic minorities, including African Americans (AAs) and Hispanics compared to NHWs, whether the treatment is for acute pain, chronic pain, cancer pain, and or palliative pain care [5], [6]. Furthermore, among AAs compared to NHWs, disability due to chronic pain is greater [7–9], quality of life among AAs with chronic knee and hip pain is poorer [6], and comorbid health conditions, including pain related anxiety, depression, and decreased physical function, are more common [6], [10], [11].

Quantitative sensory testing (QST) refers to a standardized assessment of pain sensitivity using precisely controlled protocols that elicit pain using thermal-, mechanical- or other noxious-stimuli [12]. QST measures reveal greater pain sensitivity in subjects with clinical pain compared to pain-free controls [2], [4], [13], [14]. Greater pain sensitivity to noxious stimuli is reported in AAs and Asians compared to NHWs [2], [15], [16], suggesting that experimental pain sensitivity may account for the differences in severity of clinical pain conditions among racial/ethnic groups [2].

The same pain regulatory systems that regulate QST responses have also been linked to cardiovascular function. This includes the well-established relationship between baroreceptor function and hypoalgesia, as well as vagal nerve effects upon both systems, mediated through the brain stem [17–20]. Elevated blood pressure is one indicator of cardiovascular function that is associated with decreased acute pain sensitivity (hypoalgesia), in both hypertensive and normotensive healthy individuals [21–23]. The peripheral increased BP, which activates carotid sinus baroreceptor afferent activity, triggers pain inhibitory processes–producing hypoalgesia [22], [24]. Another measure, low-frequency (LF) heart rate variability (HRV), is positively associated with increased experimental pain sensitivity [25], [26]. Decreased baroreflex sensitivity (reflecting lower parasympathetic activity) is associated with increased pain sensitivity [27]. Some evidence suggests that these cardiovascular measures are differently related to pain sensitivity across ethnic groups. For example, higher blood pressure is associated with lower pain sensitivity among NHWs, but not AAs [28]. Thus, differences in the association between cardiovascular response and pain sensitivity may relate to racial/ethnic differences in pain sensitivity.

It is likewise important to evaluate the role of psychological factors when investigating racial/ethnic differences in experimental pain sensitivity. Several psychological measures are associated with both pain sensitivity and race/ethnicity. As one example, psychological distress (e.g., anxiety, depression) has been associated with an individual’s pain, such that greater distress is associated with higher pain sensitivity [29]. Previous studies report that psychological status, including acculturation, which refers to changes that occur from contacts with culturally different people, groups, and social influences [30], [31], pain catastrophizing [32–34], coping [35], [36], and psychological distress [6], [11], [37–41], is significantly associated with racial/ethnic differences in pain sensitivity. More generally, higher levels of psychological distress occur in racial/ethnic minorities [6], [11], [37], [38], [42], and this is associated with higher pain sensitivity in racial/ethnic minorities compared to NHWs [32–34], [40], [41].

Collectively, the aforementioned studies suggest a role for psychological and cardiovascular factors, but the specific mechanisms underlying racial/ethnic differences in pain sensitivity remain unclear. The purpose of this study is to evaluate mediation effects of psychological status and cardiovascular responsiveness to racial/ethnic differences in multiple types of experimental pain sensitivity.

## Materials and methods

This secondary analysis used data from the Orofacial Pain Prospective Evaluation and Risk Assessment Study (OPPERA). The OPPERA study was originally reviewed and approved by the Institutional Review Boards at the data coordinating center and at each of the four study sites. Exemption review with minimal risk was approved from the University of Maryland IRB for the present study.

A cross-sectional study design utilized baseline data collected from 3,159 individuals (NHW: 1,637, AA: 1,012, Asian: 299, and Hispanic: 211) who did not have painful temporomandibular disorder (TMD) when enrolled in the OPPERA study. The study population, sampling methods, and baseline data collection methods have been described in detail elsewhere [43]. In summary, participants were recruited between May 2006 to November 2008 from communities in and around academic health centers at 4 US study sites (Baltimore, MD; Buffalo, NY; Chapel Hill, NC; and Gainesville, FL). Participants had the following characteristics: aged 18 to 44 years, fluent in written and spoken English, not receiving orthodontic treatment, not pregnant or nursing, negative responses to each of 10 questions about significant medical conditions (e.g., kidney failure, heart disease, chronic respiratory disease, and diabetes), TMD-free when examined by trained examiners, and no recent history of facial injury or surgery.

Race/ethnicity was assessed using a self-report question during a screening interview: “What is your race or ethnic group?”. Participants were allowed to select Hispanic and/or one or more of the following races: NHW, AA, Asian, Native Hawaiian or Other Pacific Islander, American Indian or Alaskan Native. For this analysis, people were given a single classification of race/ethnicity according to the following criteria: 1) people reporting Hispanic ethnicity were classified as Hispanic, regardless of their reported race; 2) otherwise, if a single racial group of white, African American, or Asian was reported, the person was classified as such; 3) all other people were classified as other race, multiple races or unstated, and excluded from this analysis. The reason to exclude these other groups was their small sample sizes (Pacific Islander: n = 9, Native American: n = 16, Other or multiple races: n = 65, and Unstated: n = 46).

Three modalities of noxious stimuli were tested using QST procedures—pressure pain, mechanical cutaneous pain, and heat pain—as described in detail elsewhere [14]. Briefly, pressure pain threshold (PPT) was assessed in five body sites (overlying the temporalis muscle, masseter muscle, temporomandibular joint, trapezius muscle, and flexor carpi ulnaris) bilaterally using a pressure algometer (Somedic; Horby, Sweden). For mechanical cutaneous pain sensitivity, weighted probes (2mm diameter; 8–512 mN forces) were applied to the dorsum of digits 2, 3, and 4 of the left hand. The measurements included pain threshold, suprathreshold pain intensity ratings to a single stimulus and to a series of 10 stimuli, and aftersensation ratings 15 and 30 seconds after the series of 10 stimuli. Temporal summation was derived by subtracting the ratings of a single stimulus from the ratings of a series of 10 stimuli. Both the probe set and protocol matched that used by the German Neuropathic Pain Network [44]. Contact heat stimuli were applied to the ventral forearm using the Pathway thermal stimulator (Medoc; Ramat Yishai, Israel). Measures included heat pain threshold, tolerance, single stimulus pain intensity ratings for each of 10 stimuli in a row and thermal aftersensations (ratings 15 and 30 sec after the series of 10 stimuli). The sum of 10 ratings for a given series was calculated as the aread under the curve (AUC), reflecting global heat pain sensitivity. Temporal summation of heat pain was measured in two ways: 1) the difference in rating between the highest of the 10 ratings and the first rating in the series, and 2) the slope of the regression line fit to the ratings of the first three stimuli in the series of 10 [14]. The suprathreshold ratings, aftersensations, and temporal summation measures were collected separately for stimulus intensities of 46°, 48°, and 50°C.

Cardiovascular responsiveness was measured in 5 stages: 1) a rest period (20 minutes) that followed a standardized physical examination; 2) an orthostatic challenge (5 minutes) immediately following stage 1; 3) a rest period (10 minutes) that followed assessments of thermal and mechanical pain sensitivity; 4) a traditional Stroop Color-Word test period (5 minutes) immediately following stage 3; and 5) a Stroop Pain-Affect test period (5 minutes) immediately following stage 4. At the beginning of the stage 1, a blood pressure cuff was placed on the upper left arm and was inflated with a Datascope Accutorr Plus blood pressure monitor (Datascope Inc, Mahwah, NJ). Blood pressure (BP), including systolic (SBP), diastolic (DBP), and mean (MAP) pressures, and HR were assessed every 2.5 minutes during the 20-minute rest period. During the orthostatic period, BP and HR were recorded within 30 seconds of standing and thereafter at 1-minute intervals for the next 5 minutes. Throughout the remaining three stages, BP and HR were assessed every 1-minute. MAP was calculated using the following formula: MAP = (SBP+2*DBP)/3. The ratio of HR/MAP was computed as a measure of baroreflex set point [45], the relative balance of cardiosympathetic versus cardioparasympathetic (vagal) tone. Higher values reflect greater cardiosympathetic versus vagal tone [26].

HRV, the oscillation in the interval between consecutive heartbeats, was assessed with a 3- lead BioCom model 3000 Heart Rhythm Scanner (Biocom Technologies. Poulsbo, WA). Two time domain HRV measures, SDNN (Standard deviation of normal-to-normal [N-N] intervals) and RMSSD (root-mean-square of the differences between successive N-N intervals), and three frequency domain HRV measures, VLF (very low frequency), LF (low frequency), and HF (high frequency), along with TP (total power), were calculated. The detailed methodology for cardiovascular measures is described elsewhere [26]. The assessment time for QST procedures and cardiovascular responsiveness was 1.5–2 hours.

A battery of psychological questionnaires was administered before the baseline clinical visit via paper form or electronic PDF version. Depression was measured using the 13-item depression subscale of the SCL 90R. Responses were made using a 5-point ordinal scale (not at all, a little bit, moderately, quite a bit, extremely). Internal consistency and validity for the subscale has been demonstrated [46], [47]. Internal consistency of the present study sample was good, with Cronbach’s alphas of 0.87 (NHWs), 0.90 (AAs), 0.92 (Asians), and 0.87 (Hispanics) demonstrating equal test functioning across the groups.

Anxiety was measured using the State-Trait Anxiety Inventory (STAI), which includes two 20-item questionnaires, one each for state anxiety (how they “feel right now”) and trait anxiety (how they “generally feel”). Responses were made using a 4-point ordinal scale (not at all, somewhat, moderately so, extremely so). Test-retest reliability for the Trait subscale ranges from .73 to .86, and for the State subscale from .65 to .75; with lower reliability for State presumably because of the transitory nature of anxiety states [48]. Internal consistency in the sample of each racial/ethnic group for both scales was high. Cronbach’s alpha for Trait Anxiety was 0.91 (NHWs), 0.92 (AAs), 0.91 (Asians), and 0.88 (Hispanics), and for State Anxiety 0.93 (NHWs), 0.92 (AAs), 0.93 (Asians), and 0.92 (Hispanics).

The Perceived Stress Scale (PSS) measures the degree to which individuals appraise situations as stressful and the extent to which individuals perceive themselves capable of coping with the situations. Participants are asked to indicate how often they felt stressed in the past month for each of 10 items, using a 5-point ordinal scale (never, almost never, sometimes, fairly often, very often). The total scores are computed by summing the responses to each item. Construct validity of the scale has been tested [49]. Internal consistency of the scale in the present sample was good, with Cronbach’s alphas of 0.87 (NHWs), 0.84 (AAs), 0.87 (Asians), and 0.82 (Hispanics).

The Coping Strategies Questionnaire-Revised (CSQ-R) is a revised version of the original CSQ [50], and is comprised of 27 items asking inquiring into the frequency of engagement in specific coping activities when experiencing pain using a 7-point ordinal scale, ranging from 0 (never do that) to 6 (always do that), where higher score indicating better coping. There are six subscales of pain coping strategies: diverting attention, catastrophizing, praying and hoping, ignoring pain sensations, reinterpreting pain sensations, reinterpreting pain sensations, and coping self-statements. The Catastrophizing subscale is identical to the Helplessness scale from the Pain Catastrophizing Scale (PCS). Therefore, we excluded the Catastrophizing subscale from the analyses. Previously, the stable factor structure of the scale was reported in both people with chronic pain and healthy populations [50], [51], as well as for diverse racial/ethnic groups [35]. The internal consistency of the present study sample was good, with Cronbach’s alphas of 0.88 (NHWs), 0.92 (AAs), 0.89 (Asians), and 0.88 (Hispanics).

PCS consists of 13 items rated on a 5-point ordinal scale, ranging from 0 (not at all) to 4 (all the time). Participants are asked to indicate the degree to which they have specified feelings and thoughts when experiencing pain. The instrument measures three dimensions of catastrophizing: rumination, magnification, and helplessness. A total score is calculated by summing the three subscales. The PCS has been validated for both clinical and healthy individuals [52], [53]. Also, the PCS has been previously tested in various racial/ethnic groups [33] and validation findings are acceptable. Internal consistency estimates in the present study sample were very good, with Cronbach’s alphas of 0.93 (NHWs), 0.94 (AAs), 0.94 (Asians), and 0.92 (Hispanics).

### Statistical analyses

The details of missing data handling are described elsewhere [14], [26], [54]. Briefly, the missing items on pain sensitivity, cardiovascular responsiveness and psychological status measures were imputed using expectation-maximization algorithm. Missingness on demographic variables was managed in the final models with full information maximum likelihood estimation method used by Mplus procedures. Descriptive analysis was conducted to check normality of the data. Among the HRV measures, TP, LF, VLF, and HF were log transformed because of high skewness. Principal component analysis (PCA) was conducted separately for each of the three types of data to reduce the number of variables of pain sensitivity, cardiovascular responsiveness, and psychological status. Several rotation methods (e.g., promax or varimax) were conducted to compare the interpretability of the resulting PCA loadings. Extracted components were used in subsequent multivariable analyses to examine racial/ethnic differences before testing mediation. Model fit indices were calculated using confirmatory factor analysis (CFA) with the factors forced to be the same as PCA.

#### PCA models

Heat pain threshold and tolerance, pressure pain threshold, and mechanical cutaneous pain threshold were not included in the PCA model, but instead they were analyzed separately because we have observed [15] specific patterns of racial/ethnic differences for these pain sensitivity measures. We investigated these measures separately in our analyses in order to find possible mechanisms (models) that may account for these previously described measure-specific variations. The other 27 QST measures (i.e., suprathreshold pain intensity ratings, aftersensation ratings, and temporal summation measures for mechanical and thermal stimuli) were included in a PCA analysis for data reduction. Forty derived measures of cardiovascular function were included in a separate PCA analysis. HR/MAP and all the change scores (e.g., Orthostatic ΔSBP) were not included in the PCA analysis to avoid multicollinearity as individual variables were entered separately in the model. For psychological status measures, all 12 variables selected for this study were included in another PCA analysis.

#### Process of mediation model building

First, descriptive analyses of the extracted components from PCA models were conducted; two of the pain sensitivity components (Heat Pain Aftersensations and Mechanical Cutaneous Pain Ratings /Aftersensations) were skewed and log transformed. Next, racial/ethnic differences in pain sensitivity, psychological status, and cardiovascular responsiveness were tested to find candidate variables for the final model. Linear mixed models nested by study site were used to test racial/ethnic differences. Gender, age, BMI, and education and income level were included as covariates in the analyses. Bonferroni’s method was used to adjust *p-*values for multiple comparisons to control Type I error.

Second, PCA components that showed significant differences among racial/ethnic groups were selected for further mediation analyses. A mediating variable conveys the effect of an antecedent variable on to an outcome variable, providing more detailed understanding of relations among variables [55]. The exogenous variable of our mediation models is race/ethnicity, which is a precedent to the potential mediation variables of interest. Final models were built using structural equation modeling (SEM) with full information maximum likelihood estimation method. One of the advantages of using SEM is that multiple variables can be used in a single analysis [56]. All components that showed significant racial/ethnic differences were entered into the final models. Whether observed patterns in the data are compatible with the proposed mediation hypotheses was tested using SEM. We used the following guidelines to indicate good model fit: RMSEA < 0.08, CFI ≥ 0.95, and SRMR ≤ 0.08 [57]. The non-parametric, bias-corrected 95% bootstrap confidence interval (BCI) method was used for inferential tests of the indirect effects in mediation analysis [58]. Five thousand replications created the bootstrap sample for estimating the indirect effect of mediation analysis. If a bootstrap 95% BCI does not includes 0, then the indirect effect is considered significant. Age, gender, BMI, study site, and education and income level were controlled in the mediation model. All analyses were performed using STATA 13.1 (StataCorp, Texas, USA) and Mplus 7.2 (Muthén & Muthén, Los Angeles, CA).

## Results

Demographic characteristics of the four racial/ethnic groups are shown in Table 1.

**Table 1:** Demographics of the participants.

(N = 3.159)					
		non-Hispanic white (N = 1,637)	African American (N = 1,012)	Asian (N = 299)	Hispanic (N = 211)
Age	Mean (±SD)	25.4 (6.86)	31.0 (8.68)	24.3 (5.13)	23.6 (5.38)
BMI	Mean (±SD)	25.1 (5.18)	28.9 (7.37)	23.3 (4.02)	25.8 (5.17)
Gender, no (%)	Male	708 (43.3)	422 (41.7)	135 (45.2)	84 (39.8)
	Female	929 (56.8)	590 (58.3)	164 (54.9)	127 (60.2)
Site, no (%)	NC	500 (30.5)	198 (19.6)	59 (19.7)	34 (16.1)
	NY	466 (28.5)	173 (17.1)	112 (37.5)	28 (13.3)
	FL	531 (32.4)	100 (9.9)	90 (30.1)	132 (62.6)
	MD	140 (8.6)	541 (53.5)	38 (12.7)	17 (8.1)
Health insurance	Yes	1440 (89.5)	623 (65.2)	249 (86.5)	168 (81.6)
	No	169 (10.5)	332 (34.8)	39 (13.5)	38 (18.5)
Marital Status	Married	286 (17.5)	95 (9.5)	39 (13.3)	29 (13.9)
	Living as married	57 (3.5)	54 (5.4)	4 (1.4)	7 (3.4)
	Divorced	58 (3.6)	66 (6.2)	5 (1.7)	6 (2.9)
	Separated	19 (1.2)	54 (5.4)	2 (.7)	2 (1.0)
	Widowed	3 (0.2)	5 (0.5)	0 (.0)	0 (.0)
	Never married	1200 (73.6)	710 (71.2)	243 (82.9)	163 (78.0)
	Refused	8 (0.5)	13 (1.3)	0 (.0)	2 (1.0)
Education	less than 8 yrs	1 (.1)	8 (.8)	1 (.3)	0 (.0)
	8–11 yrs	21 (1.3)	149 (15.1)	0 (.0)	5 (2.4)
	12 yrs or completed high school	96 (5.9)	263 (26.7)	15 (5.1)	11 (5.3)
	Post high school training other than college	16 (1.0)	37 (3.8)	4 (1.4)	2 (1.0)
	Some college	715 (43.8)	358 (35.4)	82 (28.0)	112 (54.1)
	College graduate	500 (30.6)	123 (12.5)	117 (39.9)	55 (26.6)
	Post graduate level	282 (17.3)	47 (4.8)	74 (25.3)	22 (10.6)
Current income	$0-$19,999	184 (13.8)	245 (34.4)	36 (18.6)	30 (18.0)
	$20,000-$39,333	251 (18.9)	230 (32.3)	58 (29.9)	30 (18.0)
	$40,000-$59,999	206 (15.5)	113 (15.9)	33 (17.0)	29 (17.4)
	$60,000-$79,999	169 (12.7)	52 (7.3)	21 (10.8)	22 (13.2)
	$80,000-$99,999	159 (12.0)	28 (3.9)	16 (8.3)	18 (10.8)
	$100,000-$149,999	187 (14.1)	32 (4.5)	14 (7.2)	25 (15.0)
	$150,000 or higher	175 (13.2)	13 (1.8)	16 (8.3)	13 (7.8)
Lifetime US residency	Yes	1503 (92.0)	926 (91.7)	81 (27.6)	103 (49.3)
	No	130 (8.0)	84 (8.3)	213 (72.5)	106 (50.7)
#of yrs in the US	Mean (±SD)	19.6 (10.76)	26.7 (12.86)	8.5 (8.59)	13.5 (7.72)
First Spoken	Yes	1563 (95.7)	981 (97.7)	103 (35.5)	77 (36.8)
Language (English)	No	70 (4.3)	23 (2.3)	187 (64.5)	132 (63.2)

### PCA results

#### Pain sensitivity

**Fig 1:**
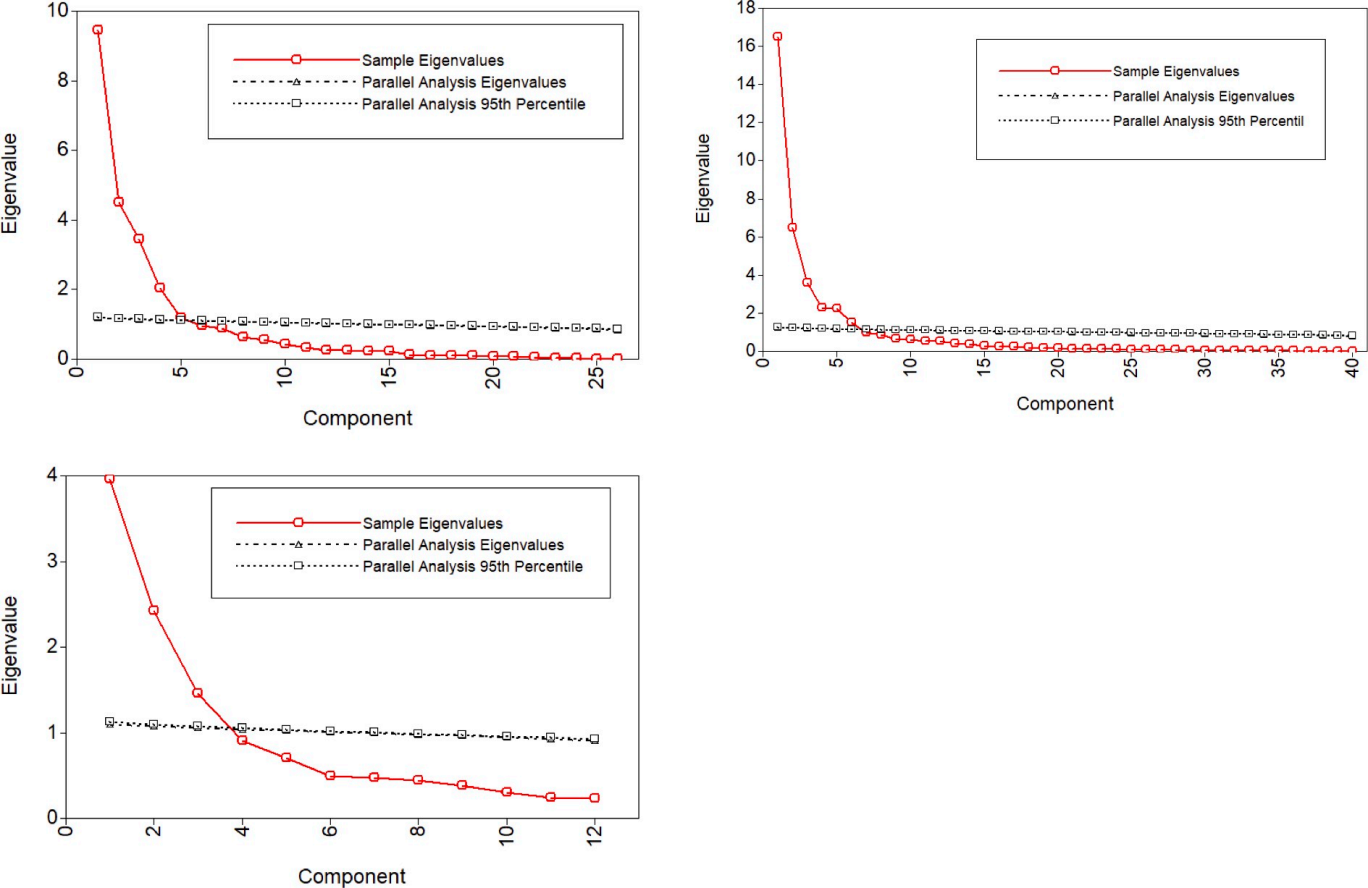
Components resulting from principal component analysis (red lines) and parallel analysis (black lines). Note. Upper left: pain sensitivity data, Upper right: cardiovascular responsiveness data, Bottom: psychological status data.

The number of retained principal components was determined taking into account the interpretation of the patterns, percentage of total variances explained and the visual inflections in the scree plot as well as the parallel analysis. Scree plots display the variances (eigenvalue) for each component extracted from the data. As shown in Fig 1 (upper left panel), the variances explained by each principal component decreased most noticeably after the fifth component, indicating that first 5 components contributed substantially in explaining the variances of the data. This estimate was supported with a parallel analysis with 1000 simulations [59]. A final solution of five components explained 75.9% of the variance. Varimax rotations increased the interpretability of the resulting PCA loadings (S1 File). The first component, accounting for 20% of the variance, was labeled Heat Pain Aftersensation (HPA) and includes high loadings from pain ratings given 15 and 30 seconds post-stimulation. The second component, accounting for an additional 18% of the variance, was labeled Heat Pain Rating (HPR) and includes high loadings from single (first) stimulus pain ratings and area under the curve (AUC) of ratings from multiple heat stimuli. The third component, accounting for an additional 15% of the variance, was labeled Heat Pain Temporal Summation (HPTS) and includes high loadings for two such measures: 1) delta heat pain ratings from maximum pain rating–first rating, and 2) slope of the regression line of the first three heat pain ratings. The fourth component, accounting for an additional 12% of the variance, was labeled Mechanical Cutaneous Pain Ratings and Aftersensations (MCPRA) and includes high loadings from single stimulus mechanical pain ratings, and aftersensations of mechanical pain stimuli. The fifth component, accounting for an additional 11% of the variance, was labeled Mechanical Cutaneous Pain Temporal Summation (MCPTS) and includes high loadings from mechanical temporal summation measured derived from two stimulus intensities. Although the eigenvalue of the fifth component was just over 1, this component was retained because of better interpretability of the resulting loadings. The Cronbach’s alpha values for these five components were high, ranging from .80 to .96, indicating good reliability of this model. Model fit from CFA indicated an adequate model fit: RMSEA = .079, CFI = .950, and SRMR = .070.

#### Cardiovascular responsiveness

Scree plots and parallel analysis suggested first 6 components explained the most variances (Fig 1, Upper right); however, the interpretability of the resulting loadings was poor, even after several rotation methods were applied. For example, the loading difference between the highest and next highest components of both Stroop SNDD and TP was less than .100. Therefore, we refit the PCA model using the five strongest components, and the PCA loading interpretability with varimax rotation improved over the 6-component model (S2 File). In total, 76% of the variance was explained by the five components. The first component, accounting for 22% of the variance, was labeled Stroop HRV and includes high loadings from both time and frequency domains collected during both Stroop Color-Word and Pain Affect tests. The second component, accounting for an additional 18% of the variance, was labeled Blood Pressure (BP) and includes high loadings from SBP, DBP, and MAP obtained during Rest and Stroop tests. The third component, accounting for an additional 15% of the variance, was labeled Heart Rate (HR), and includes high loadings for measures of HR across all protocols (Rest, Orthostatic, and Stroop). The fourth component, accounting for an additional 11% of the variance, was labeled Baseline HRV and includes high loadings from both time and frequency HRV domains collected during the resting period. The fifth component, accounting for an additional 10% of the variance, was labeled Orthostatic HRV and includes high loadings from both time and frequency HRV domains collected during the orthostatic challenge period. The Cronbach’s alpha values for these five components were high–ranging from .89 to .96 –indicating good reliability of this model. Model fit from CFA indicated an adequate model fit: RMSEA = .079, CFI = .930, and SRMR = .070.

#### Psychological status

As shown in Fig 1 (Bottom panel), PCA models extracted three principal components which explained 65.1% of the variance. Promax rotations increased the interpretability of the resulting PCA loadings (S3 File). The first component, accounting for 33% of the variance, was labeled Depression, Anxiety, and Stress (DAS) and includes high loadings from the STAI, the PSS, and the depression subscale of the SCL-90. The second component, accounting for an additional 20% of the variance, was labeled Pain Catastrophizing (PC) and includes high loadings from the rumination, magnification, and helplessness subscales of the PCS, and the praying subscale of the CSQ. The third component, accounting for an additional 12% of the variance, was labeled Pain Coping and includes high loadings for the remaining subscales of CSQ: distraction, distancing, ignoring, and coping self-statement. The Cronbach’s alpha values for these three components were acceptable (ranged from .73 to .89). Model fit from CFA indicated an adequate model fit: RMSEA = .065, CFI = .962, and SRMR = .057.

### Racial/Ethnic differences in pain sensitivity

Among the PCA components of pain sensitivity, NHWs provided lower heat pain ratings than AAs (*β* = 1.000 p < .001), Asians (*β* = .950, p < .001), and Hispanics (*β* = .717, p < .001; Table 2). AAs and Asians provided higher ratings than NHWs in HPA (*β* = .172, p < .001; *β* = .123, p = .001, respectively), in MCPRAS (*β* = .151, p < .001; *β* = .294, p < .001, respectively), and in MCPTS (*β* = .365, p < .001; *β* = .698, p < .001, respectively). Among the individually evaluated variables, no significant racial/ethnic differences were found for heat pain threshold, PPT or HPTS. But, AAs and Asians, compared to NHWs, had lower heat pain tolerance (*β* = -.632, p < .001; *β* = -1.009, p < .001, respectively). Asians had lower mechanical cutaneous pain thresholds than did NHWs (*β* = -53.501, p < .001).

**Table 2:** Linear mixed models to test racial/ethnic differences in pain sensitivity.

		Coef.	SE	z	p
Heat pain ratings (PCA Comp.1)					
	African Americans	1.000	.128	7.83	**< .001**
	Asians	.950	.176	5.41	**< .001**
	Hispanics	.717	.180	3.98	**< .001**
Heat pain aftersensation (PCA Comp.2, log transformed)					
	African Americans	.172	.028	6.18	**< .001**
	Asians	.123	.038	3.20	**.001**
	Hispanics	.085	.039	2.16	.031
Heat pain temporal summation (PCA Comp.3)					
	African Americans	.131	.126	1.04	.298
	Asians	.188	.174	1.08	.280
	Hispanics	.140	.178	.78	.434
Mechanical cutaneous pain ratings and aftersensation (PCA Comp.4, log transformed)					
	African Americans	.151	.029	5.27	**< .001**
	Asians	.294	.039	7.46	**< .001**
	Hispanics	.094	.040	2.33	.020
Mechanical cutaneous pain temporal summation (PCA Comp.5)					
	African Americans	.365	.077	4.71	**< .001**
	Asians	.698	.107	6.55	**< .001**
	Hispanics	.192	.109	1.76	.079
Heat pain tolerance					
	African Americans	-.632	.126	-5.00	**< .001**
	Asians	-1.009	.175	-5.77	**< .001**
	Hispanics	-.374	.184	-2.03	.042
Heat pain threshold					
	African Americans	.218	.197	1.11	.268
	Asians	-.622	.259	-2.40	.017
	Hispanics	.080	.267	.30	.763
Pressure pain threshold					
	African Americans	3.982	4.749	.84	.402
	Asians	-10.405	6.520	-1.60	.111
	Hispanics	4.829	7.016	.69	.491
Mechanical cutaneous pain threshold					
	African Americans	-4.123	9.421	-.44	.662
	Asians	-53.501	12.826	-4.17	**< .001**
	Hispanics	-20.528	13.871	-1.48	.139

Note. PCA: Principal Component Analysis; Models were nested by study site, reference group was non-Hispanic whites, covariates: age, gender, and income & education level; numbers in bold reflect significant p-values after Bonferroni correction.

### Racial/Ethnic differences in cardiovascular responsiveness

AAs had higher BP than did NHWs (*β* = 1.215, p < .001), and Asians had higher HR compared to NHWs (*β* = .625, p = .002). Among the individually evaluated variables, there were no racial/ethnic differences in orthostatic delta SBP, DBP, MAP, or HR measures. HR/MAP index was lower in AAs compared to NHWs across all protocols. For Asians, HR/MAP index was higher compared to NHWs across all protocols. There were no significant differences between Hispanic and NHWs in any cardiovascular measure (Table 3).

**Table 3:** Linear mixed models to test racial/ethnic differences in cardiovascular responsiveness.

		Coef.	SE	z	p
Stroop Heart Rate Variability (PCA Comp.1)					
	African Americans	.333	.181	1.84	.066
	Asians	-.475	.235	-2.02	.043
	Hispanics	.010	.249	.04	.967
Blood Pressure (PCA Comp.2)					
	African Americans	1.215	.153	7.93	**< .001**
	Asians	-.207	.199	-1.04	.298
	Hispanics	-.385	.210	-1.83	.067
Heart Rate (PCA Comp.3)					
	African Americans	.143	.152	.94	.349
	Asians	.625	.198	3.15	**.002**
	Hispanics	.278	.210	1.32	.186
Baseline HRV (PCA Comp.4)					
	African Americans	-.232	.129	-1.80	.073
	Asians	-.426	.168	-2.54	.011
	Hispanics	-.179	.178	-1.01	.313
Orthostatic HRV (PCA Comp.5)					
	African Americans	-.206	.130	-1.59	.113
	Asians	-.312	.169	-1.85	.064
	Hispanics	.220	.179	1.23	.217
Orthostatic ΔSBP (first Orthostatic period SBP—resting mean SBP)					
	African Americans	-.802	.796	-1.01	.314
	Asians	.238	1.070	.22	.824
	Hispanics	.414	1.157	.36	.720
Orthostatic ΔDBP (first Orthostatic period DBP—resting mean DBP)					
	African Americans	-.096	.523	-.18	.854
	Asians	-1.170	.702	-1.67	.096
	Hispanics	-.363	.761	-.48	.634
Orthostatic ΔMAP (first Orthostatic period MAP—resting mean MAP)					
	African Americans	-.175	.539	-.33	.745
	Asians	-.406	.722	-.56	.574
	Hispanics	-.443	.780	-.57	.571
Orthostatic ΔHR (first Orthostatic period HR—resting mean HR)					
	African Americans	-1.318	.791	-1.67	.095
	Asians	-3.008	1.063	-2.83	.005
	Hispanics	-2.457	1.153	-2.13	.033
Baseline HR/MAP ratio					
	African Americans	-.030	.007	-4.26	**< .001**
	Asians	.032	.009	3.37	**.001**
	Hispanics	.024	.010	2.35	.019
Color-Word Stroop HR/MAP ratio					
	African Americans	-.027	.007	-3.74	**< .001**
	Asians	.034	.009	3.57	**< .001**
	Hispanics	.022	.010	2.16	.031
Pain-Affect Stroop HR/MAP ratio					
	African Americans	-.026	.007	-3.59	**< .001**
	Asians	.040	.010	4.07	**< .001**
	Hispanics	.014	.010	1.34	.179

Note. PCA: Principal Component Analysis; Models were nested by study site, reference group was non-Hispanic whites, covariates: age, gender, BMI, and income & education level; numbers in bold reflect significant p-values after Bonferroni correction

### Racial/Ethnic differences in psychological status

Asians had higher levels in DAS than did NHWs (*β* = .449, p < .001). AAs (*β* = .520, p < .001) and Asians (*β* = .548, p < .001) had higher levels of PC compared to NHWs (Table 4). Coping was also higher in racial/ethnic minorities; AAs, Asians, and Hispanics had high levels of coping than did NHWs (*β* = .445, p < .001; *β* = .489, p < .001; *β* = .352, p = .005, respectively).

**Table 4:** Linear mixed models to test racial/ethnic differences in psychological status.

		Coef.	SE	z	p
Depression, Anxiety, and Stress (PCA Comp.1)					
	African Americans	.021	.091	-.23	.816
	Asians	.449	.125	3.59	**< .001**
	Hispanics	-.055	.135	-.41	.681
Pain catastrophizing (PCA Comp.2; rumination, magnification, helplessness, and praying)					
	African Americans	.520	.088	5.91	**< .001**
	Asians	.548	.121	4.54	**< .001**
	Hispanics	.129	.130	.00	.321
Coping (PCA Comp.3; distraction, distancing, ignoring, and coping self-statements)					
	African Americans	.445	.085	5.22	**< .001**
	Asians	.489	.117	4.18	**< .001**
	Hispanics	.352	.126	2.79	**.005**

Note. PCA: Principal Component Analysis; Models were nested by study site, reference group was non-Hispanic whites, covariates: age, gender, and income & education level; numbers in bold reflect significant p-values after Bonferroni correction.

### Final mediation models

Separate mediation model analyses were conducted to evaluate QST differences between AAs–NHWs, Asians–NHWs, and Hispanics–NHWs. Pain sensitivity measures that showed significant racial/ethnic differences (AAs vs. NHWs—heat pain tolerance, HPR, HPA, MCPRAS, and MCPTS; Asians vs. NHWs—heat pain tolerance, mechanical cutaneous pain threshold, HPR, HPA, MCPRAS, and MCPTS; Hispanics vs. NHWs—HPR) were selected as outcome variables in the final models. All of the cardiovascular and psychological measures that showed significant racial/ethnic differences were entered into the final mediation model for each group. Therefore, BP, HR/MAP index, PC, and Coping were tested for their mediation effects on racial/ethnic differences in pain sensitivity between AAs and NHWs. HR, HR/MAP index, DAS, PC, and coping were entered for Asians to test mediation effects on racial/ethnic differences in pain sensitivity compared to NHWs. Coping was entered for Hispanics to test mediation effects on the racial/ethnic differences in pain sensitivity compared to NHWs. Age, gender, BMI, study site, and education and income level were entered in the models simultaneously as covariates.

#### AAs versus NHWs

**Fig 2:**
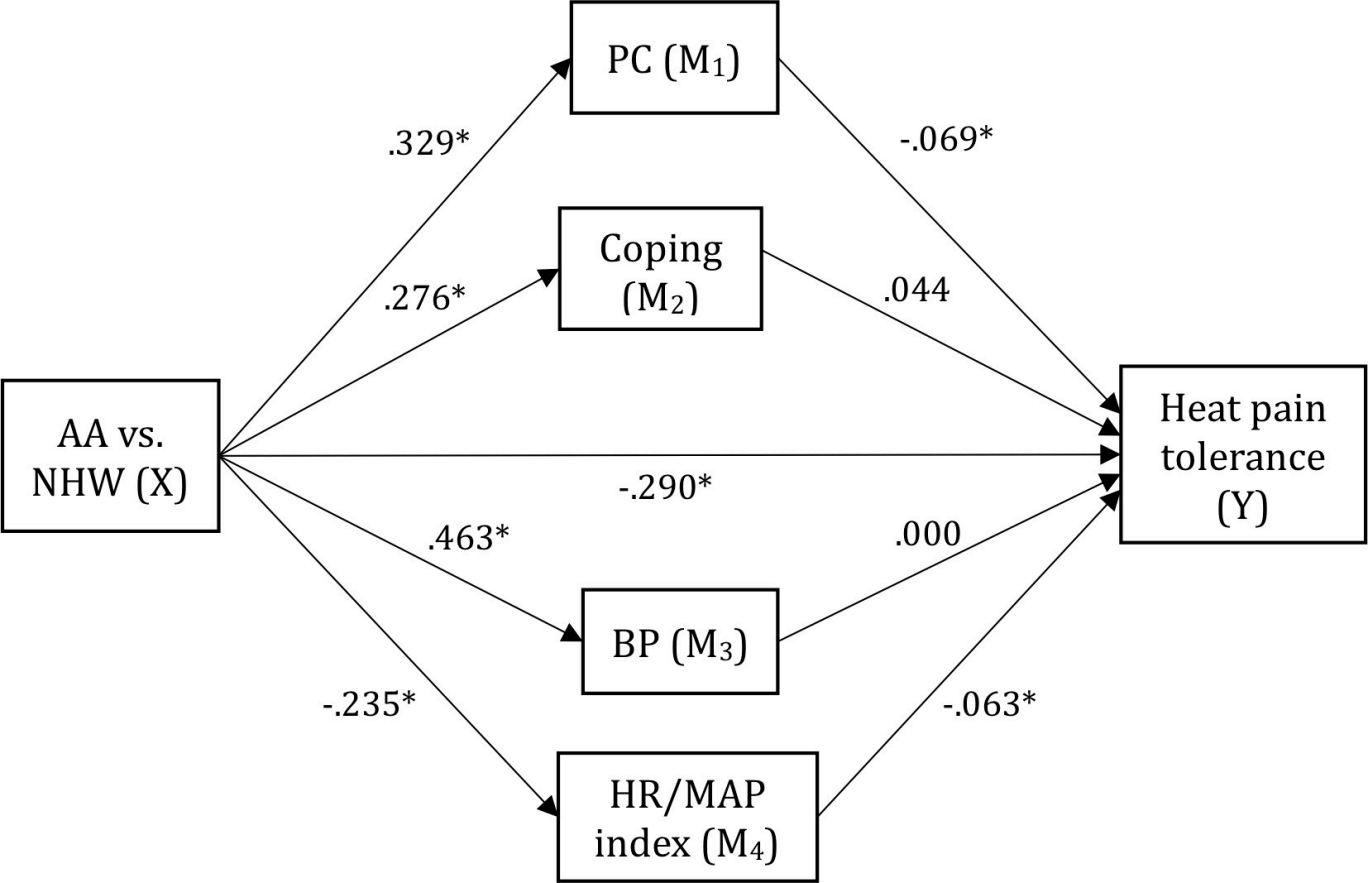
Mediation model for AA-NHW differences in heat pain tolerance. Note. Pain Catastrophizing (PC) partially mediated the association between AA-NHW differences in heat pain tolerance, indicating that AA-NHW differences in heat pain tolerance were partially explained by higher PC in AAs; HR/MAP index negatively mediated the association, indicating that lower HR/MAP index (higher baroreflex set point) in AAs partially suppressed AA-NHW differences in heat pain tolerance. Path values represent standardized beta coefficients (*p < .05). Full statistics are presented in Table 5. The models estimated in the Figure were:
$$M_{k}=a_{0}+a_{k}X+covariates+\varepsilon_{k};\;k=1,2,\ldots ,4$$
$$Y=b_{0}+b_{k}M_{k}+c^{\prime }X+covariates+\varepsilon ;\;k=1,2,\ldots ,4$$
Where age, gender, BMI, study site, and education and income level were entered as covariates. The product of *a_k_b_k_* represents the indirect effect for k^th^ mediator and c’ the direct effect.

PC was a significant mediator for AA-NHW differences in heat pain tolerance, HPR, HPA, MCPRAS, and MCPTS (Table 5). With respect to heat pain tolerance, HR/MAP index showed a significant “inconsistent” mediating effect (Fig 2). Coping also showed a significant inconsistent mediating effect for AA-NHW differences in MCPTS (Table 5).

**Table 5:** Multiple mediation models (African Americans vs. non-Hispanic whites).

	Standardized Coefficient	s.e.	p	Bootstrap 95% CI
*Heat pain tolerance*				
Direct effect (c' path)	-.290	.056	< .001	**-.389, -.169**
Indirect effect (via mediators)				
PC	-.023	.009		**-.043, -.008**
Coping	.012	.007		-.001, .027
BP	.000	.013		-.026, .027
HR/MAP index	.015	.007		**.003, .033**
*Heat pain ratings*				
Direct effect (c' path)	.204	.029	< .001	**.309, .547**
Indirect effect (via mediators)				
PC	.018	.005		**.018, .059**
Coping	-.007	.003		-.029, .000
BP	.010	.007		-.008, .051
HR/MAP index	-.002	.003		-.018, .010
*Heat pain aftersensations*				
Direct effect (c' path)	.350	.069	< .001	**.215, .485**
Indirect effect (via mediators)				
PC	.037	.011		.**015, .059**
Coping	-.015	.008		-.030, .000
BP	.016	.016		-.015, .047
HR/MAP index	-.005	.007		-.019, .008
*Mechanical cutaneous pain ratings and aftersensations*				
Direct effect (c' path)	.327	.070	< .001	**.190, .463**
Indirect effect (via mediators)				
PC	.042	.012		**.019, .065**
Coping	-.001	.007		-.015, .013
BP	-.009	.015		-.039, .021
HR/MAP index	-.006	.007		-.020, .008
*Mechanical cutaneous pain temporal summation*				
Direct effect (c' path)	.299	.074	< .001	**.154, .444**
Indirect effect (via mediators)				
PC	.030	.010		**.011, .049**
Coping	-.017	.008		**-.032, -.003**
BP	.002	.015		-.028, .032
HR/MAP index	-.001	.007		-.015, .013

Note. PC: pain catastrophizing; BP: blood pressure; HR/MAP: heart rate/ mean arterial pressure; Covariates: age, gender, BMI, study site, and education and income level; numbers in bold reflect significant results.

#### Asians versus NHWs

**Fig 3:**
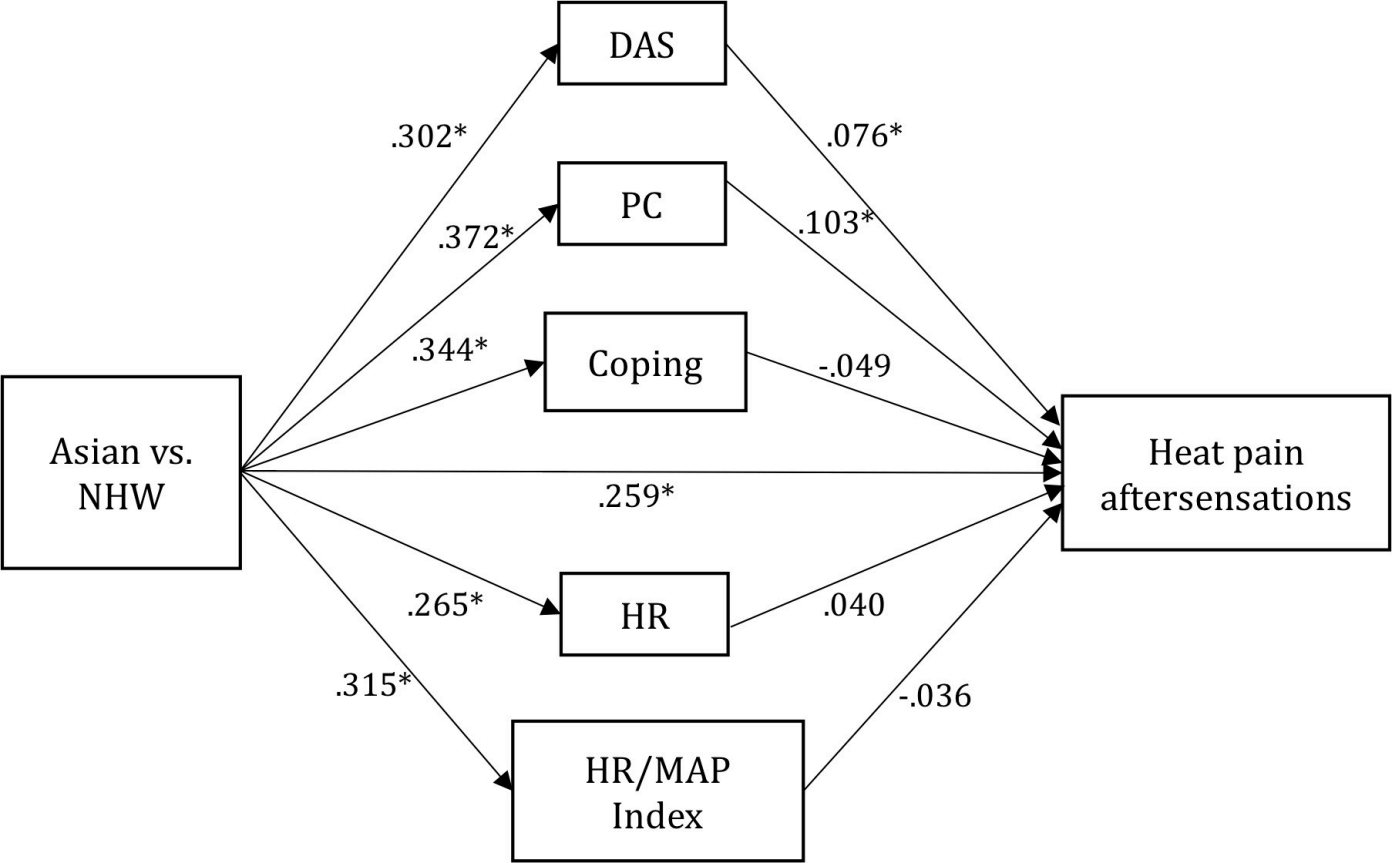
Mediation model for Asian-NHW differences in heat pain aftersensations. Note. Depression, Anxiety, and Stress (DAS) and Pain Catastrophizing (PC) partially mediated the association between Asian-NHW and heat pain ratings, indicating that Asian-NHW differences in heat pain aftersensations were partially explained by higher levels of DAS and PC in Asians. Path values represent standardized beta coefficients (*p < .05). Full statistics are presented in Table 6. Similar models as in Fig 2 were estimated. Age, gender, BMI, study site, and education and income level were entered as covariates.

PC was a significant mediator for Asian-NHW differences in heat pain tolerance, HPR, HPA, MCPRAS, and MCPTS (Table 6). DAS was another significant mediator for Asians-NHW differences in HPR. Coping showed a significant inconsistent mediating effect in Asians-NHW differences for MCPTS. The mediation model for heat pain aftersensations is seen in Fig 3 (Table 6).

**Table 6:** Multiple mediation models (Asians vs. non-Hispanic whites).

	Standardized Coefficient	s.e.	p	Bootstrap 95% CI
*Heat pain tolerance*				
Direct effect (c' path)	-.386	.080	< .001	**-.543, -.228**
Indirect effect (via mediators)				
DAS	-.010	.009		-.028, .008
PC	-.024	.012		**-.047, -.001**
Coping	.016	.010		-.003, .036
HR	.005	.014		-.022, .032
HR/MAP index	-.031	.018		-.067, .005
*Mechanical cutaneous pain threshold*				
Direct effect (c' path)	-.270	.077	< .001	**-.421, -.118**
Indirect effect (via mediators)				
DAS	-.019	.010		-.040, .001
PC	-.015	.011		-.037, .007
Coping	.017	.010		-.003, .037
HR	.002	.013		-.023, .028
HR/MAP index	-.008	.016		-.041, .024
*Heat pain ratings*				
Direct effect (c' path)	.375	.086	< .001	**.206, .544**
Indirect effect (via mediators)				
DAS	.015	.010		-.005, .035
PC	.046	.016		**.014, .078**
Coping	-.017	.011		-.038, .003
HR	.007	.014		-.020, .034
HR/MAP index	-.011	.017		-.043, .022
*Heat pain aftersensations*				
Direct effect (c' path)	.259	.100	.009	**.064, .454**
Indirect effect (via mediators)				
DAS	.023	.011		**.002, .044**
PC	.038	.016		**.007, .070**
Coping	-.017	.010		-.036, .002
HR	.010	.015		-.018, .039
HR/MAP index	-.011	.018		-.046, .023
*Mechanical cutaneous pain ratings and aftersensations*				
Direct effect (c' path)	.562	.106	< .001	**.354, .770**
Indirect effect (via mediators)				
DAS	.014	.010		-.006, .033
PC	.038	.016		**.006, .069**
Coping	-.002	.010		-.021, .018
HR	-.012	.014		-.039, .015
HR/MAP index	.029	.019		-.007, .066
*Mechanical cutaneous pain temporal summation*				
Direct effect (c' path)	.559	.104	< .001	**.355, .762**
Indirect effect (via mediators)				
DAS	.005	.009		-.012, .022
PC	.034	.014		**.007, .061**
Coping	-.027	.011		**-.050, -.005**
HR	-.010	.016		-.042, .021
HR/MAP index	.006	.017		-.027, .039

Note. DAS: depression, anxiety, and stress; PC: pain catastrophizing; HR: heart rate; Covariates: age, gender, BMI, study site, and education and income level; numbers in bold reflect significant results.

#### Hispanics versus NHWs

Coping was not found to be a significant mediator for Hispanic-NHW differences in HPR (indirect effect = -.001, Bootstrap 95% CI: -.036, .016).

## Discussion

Racial/ethnic differences in experimental pain sensitivity were found for many of our measures, and largely replicated studies that evaluated such measures individually. Pain catastrophizing was the most robust mediator of racial/ethnic differences in pain sensitivity, as it was found relevant to most pain measures for both AAs and Asians compared to NHWs. Two other psychological measures (DAS and Coping) and one cardiovascular response measure (HR/MAP) were found to be significant mediators for individual pain measures. Two of these mediators–Coping and HR/MAP–were inconsistent mediators, suggesting that they mitigated the total racial/ethnic differences in experimental pain sensitivity. Inconsistent mediation means that the mediator’s effects are in the opposite direction of the direct effects of the racial/ethnic differences in pain sensitivity, thus the total racial/ethnic differences combining the mediator’s effects and direct effects mitigated [55].

### Racial/Ethnic differences in experimental pain sensitivity

In our analyses, AAs, compared to NHWs, had lower heat pain tolerance and higher suprathreshold pain ratings (HPR, HPA, MCPRAS, and MCPTS), while no differences were found for any threshold measure or for HPTS. These results are consistent with a recent systematic review and meta-analysis of racial/ethnic differences in experimental pain sensitivity, which found significant differences in tolerance and suprathreshold pain intensity ratings but not in pain threshold between AAs and NHWs [15]. Asians, compared to NHWs, had higher suprathreshold pain ratings (HPR, HPA, MCPRAS, and MCPTS), and a lower heat pain tolerance and mechanical cutaneous pain threshold. However, it is difficult to compare these results with previous studies, due to the differences in context and settings of Asian population samples of previous studies [15].

Regarding temporal summation of pain, we found mixed results in racial/ethnic differences by pain modality in the present study. MCPTS measures were significantly different between AAs and NHWs, while HPTS measures were not. Few studies have examined racial/ethnic differences in temporal summation and results are mixed by pain modalities and participant characteristics [15].

### Pain Catastrophizing (PC), a strong mediator for racial/ethnic differences in pain sensitivity

Both AAs and Asians had higher PC scores and greater pain sensitivity in many QST measures compared to NHWs. PC was found to be a significant mediator in 10 of 11 pain sensitivity measures showing these racial/ethnic differences, including heat pain tolerance, HPR, HPA, MCPRAS, and MCPTS. At least three studies have reported similar mediating effects of PC on racial/ethnic differences in pain tolerance and pain intensity [32], [33], [60]. More recently, Meints and colleagues reported that the rumination component of catastrophizing mediated differences in cold pain tolerances between AA and NHW participants [61].

However, PC was not a significant mediator for the association between Asians-NHWs differences and mechanical cutaneous pain threshold. This is the first study reporting such differences of effects of PC among these various pain sensitivity measures. The result of the present study suggests that PC specifically mediates race/ethnicity differences for suprathreshold and affective pain responses. This idea is consistent with neuroimaging studies that show PC is associated with increased responses to noxious stimuli in brain areas related to attention to pain, anticipation of pain, and emotional aspects of pain [62], [63].

### Coping as an inconsistent mediator between race/ethnicity and pain sensitivity

The mediation effect of coping on the association between race/ethnicity and pain sensitivity for mechanical cutaneous pain temporal summation in both AAs and Asians (compared to NHWs) was inconsistent, which indicates the mediation effects of coping are in the opposite direction of the total effects of the racial difference in pain sensitivity. The inconsistent mediation effect is a type of suppressor effect [55]. This indicates that the racial differences in mechanical cutaneous pain temporal summation would be mitigated by the indirect effect of the coping. AAs and Asians reported higher coping than NHWs, and higher coping was associated with lower pain sensitivity (lower MCPTS). Higher utilization of coping strategies in AAs and Asians would decrease specific aspects of pain sensitivity to a greater extent compared to NHWs, which would ultimately decrease racial/ethnic differences in pain sensitivity.

A previous study examining mediation effect of coping reported significant mediation effects on the racial/ethnic differences (AAs vs. NHWs) in tolerance to cold pressor pain; higher scores of the two SCQ subscales—Catastrophizing and Praying—in AAs compared to NHWs were associated with a lower pain tolerance in AAs [36]. In our analyses, Coping included high loadings of the other four subscales of CSQ—Distraction, Distancing, Ignoring and Coping self-statement. The results of the present study contribute to the literature by revealing the mediating effects of these remaining subscales on the racial/ethnic differences in pain sensitivity, specifically temporal summation of mechanical cutaneous pain. This result suggests that using the pain coping strategies in pain management for patients with chronic pain might be beneficial in decreasing clinical pain, perhaps more strongly for AAs and Asians.

### Depression, Anxiety, and Stress (DAS) as a mediator for the association between race/ethnicity (Asians vs. NHWs) and pain sensitivity

Depression, anxiety, and stress are associated with higher pain sensitivity among patients with chronic pain [6], [11], [37–41]. We found positive associations between DAS and pain sensitivity in our analyses of data from healthy young adults. DAS mediated racial/ethnic differences (Asians vs. NHWs) in HPA in our analyses. Asians had higher levels of DAS compared to NHWs, and higher DAS was associated with higher heat pain aftersensation. This result is important especially for Asians in the US because higher rates of depression among Asian Americans in the community have been recently reported [42]. Recently, a study also reported the contribution of depression to clinical pain in Asians-Americans with knee osteoarthritis [64]. Levels of depressive symptoms, anxiety, and stress should be considered in developing pain management programs, and these results suggest a particularly valuable role for improving pain management outcomes for Asians in the US.

### Baroreflex setpoint as an inconsistent mediator between race/ethnicity and pain sensitivity

AAs, compared to NHWs, had a lower HR/MAP index, indicating greater parasympathetic than sympathetic tone. A negative association between HR/MAP index and heat pain tolerance was found in the present study; in other words, higher HR/MAP index (higher baroreflex setpoint) was associated with lower heat pain tolerance (hypersensitivity). This is in line with previous findings of low parasympathetic activation and high sympathetic activation (e.g., high HR/MAP index), associated with reduced pressure pain tolerance in patients with high somatic awareness [65]. Several studies have reported a negative association between baroreflex sensitivity and pain perception in no-pain and chronic pain patients [26], [66].

The mediation model to explain racial/ethnic differences in heat pain tolerance between AAs and NHWs revealed that HR/MAP index was an inconsistent mediator on the association between race/ethnicity and heat pain tolerance. This indicates that the racial differences in heat pain tolerance would be mitigated by the indirect effect of the HR/MAP index. While AAs in our sample had significantly higher BP compared to NHWs, BP alone was not found to mediate any of the pain sensitivity differences. These results suggest that the most critical factor would be parasympathetic/sympathetic balance, which was reflected in the HR/MAP index. The baroreflex response provides an immediate increase in parasympathetic activity and a decrease in sympathetic activity, following a sudden increase in blood pressure [67]. Although AAs had higher BP, the HR/MAP index suggests that they also had greater parasympathetic activity compared to NHWs. This greater parasympathetic activity in AAs appears to mitigate racial/ethnic differences in heat pain tolerance.

### Limitations

These data were derived from a convenience sample with certain exclusion criteria; therefore, generalizability of the results is limited. First, we only included racial/ethnic minorities who were fluent in English because many of the study instruments were only available in English. A language barrier is one of the important factors in pain disparity [6], [68], [69], and multi-lingual studies are needed to achieve a fuller representation of racial/ethnic minorities. Second, the participants were largely healthy adults, and results may be different for non-healthy or older populations. Based on the current evidence regarding the impact of clinical pain on pain sensitivity, future studies on patients with chronic pain conditions should examine racial/ethnic differences in pain sensitivity. Lastly, some specific measures were not evaluated directly because several analyses in this study used component scores from PCA analyses, which is indicative of relative contributions of the initial parameters.

## Conclusions

The current study provides several novel findings regarding factors significantly contributing to racial/ethnic differences in experimental pain sensitivity. The identified mediators, most notably pain catastrophizing, should be considered in pain management programs to implement better strategies to reduce clinical pain. Such better strategies may be particularly beneficial for AAs and Asians in the US. Other psychological and cardiovascular response factors appear to have very selective mediating effects, suggesting different mechanisms are relevant for racial/ethnic differences in specific types of pain. Further clinical and experimental research is required to increase our understanding of the suggested mechanisms explaining racial/ethnic differences in pain sensitivity and to extend our findings to clinical pain populations.

## Supporting information

S1 File Component loadings for principal component analysis (PCA) model for pain sensitivity. Note. Numbers in bold reflect the highest loading for each variable.
(DOCX)

S2 File Component loadings for principal component analysis (PCA) model for cardiovascular responsiveness. Note. Numbers in bold reflect the highest loading for each variable.
(DOCX)

S3 File Component loadings for principal component analysis (PCA) model for psychological status. Note. Numbers in bold reflect the highest loading for each variable.
(DOCX)
