# Supplementary material for: Racial/ethnic differences in experimental pain sensitivity and associated factors – Cardiovascular responsiveness and psychological status
Source: PLoS One. 2019 Apr 18;14(4):e0215534. doi: 10.1371/journal.pone.0215534 (PMC6472780; doi:10.1371/journal.pone.0215534)
Supplement: S1 File — Note. Numbers in bold reflect the highest loading for each variable. (DOCX) [file pone.0215534.s001.docx]

| **S1 File. Component loadings for principal component analysis (PCA) model for pain sensitivity** | | | | | |
| --- | --- | --- | --- | --- | --- |
|  | Comp. 1 | Comp. 2 | Comp. 3 | Comp. 4 | Comp. 5 |
| Single stimulus ratings |  |  |  |  |  |
| 256mN probe | .017 | .321 | -.026 | **.707** | .092 |
| 512nM probe | .074 | .392 | -.005 | **.711** | .150 |
| Aftersensation ratings |  |  |  |  |  |
| 15s, 256mN probe | .310 | .021 | .004 | **.845** | .119 |
| 30s, 256mN probe | .320 | -.023 | -.001 | **.802** | .027 |
| 15s, 512mN probe | .427 | .101 | .049 | **.768** | .187 |
| 30s, 512mN probe | .451 | .050 | .035 | **.746** | .096 |
| Temporal Summation |  |  |  |  |  |
| 256mN probe | .131 | .169 | .010 | .354 | **.821** |
| 512mN probe | .170 | .219 | .093 | .133 | **.880** |
| Single stimulus ratings |  |  |  |  |  |
| 46 °C | .147 | **.868** | -.197 | .140 | .045 |
| 48 °C | .155 | **.915** | -.165 | .125 | .106 |
| 50 °C | .158 | **.890** | -.063 | .083 | .093 |
| Ratings of 10 stimuli: area under curve | |  |  |  |  |
| 46 °C | .185 | **.865** | .262 | .145 | .104 |
| 48 °C | .199 | **.880** | .336 | .128 | .111 |
| 50 °C | .188 | **.802** | .369 | .106 | .107 |
| Heat pain aftersensations |  |  |  |  |  |
| 15s, 46 °C | **.807** | .221 | .049 | .194 | .030 |
| 30s, 46 °C | **.854** | .214 | .071 | .217 | .109 |
| 15s, 48 °C | **.845** | .159 | .075 | .214 | .112 |
| 30s, 48 °C | **.837** | .129 | .034 | .184 | .013 |
| 15s, 50 °C | **.883** | .153 | .049 | .195 | .078 |
| 30s, 50 °C | **.869** | .090 | .058 | .211 | .079 |
| Temporal summation: highest minus first rating | | |  |  |  |
| 46 °C | .063 | .049 | **.830** | -.002 | .089 |
| 48 °C | .057 | -.009 | **.908** | -.018 | -.014 |
| 50 °C | .042 | -.119 | **.858** | .001 | -.035 |
| Temporal summation: slope of line for first three ratings | | |  |  |  |
| 46 °C | .033 | .185 | **.702** | .030 | .112 |
| 48 °C | .042 | .110 | **.837** | -.003 | .000 |
| 50 °C | .034 | .008 | **.843** | .020 | -.028 |
| Cumulative variance | .20 | .38 | .53 | .65 | .76 |
| Cronbach's alpha | .94 | .96 | .91 | .90 | .80 |
| Note. Numbers in bold reflect the highest loading for each variable. | | | |  |  |
