# Supplementary material for: Racial/ethnic differences in experimental pain sensitivity and associated factors – Cardiovascular responsiveness and psychological status
Source: PLoS One. 2019 Apr 18;14(4):e0215534. doi: 10.1371/journal.pone.0215534 (PMC6472780; doi:10.1371/journal.pone.0215534)
Supplement: S2 File — Note. Numbers in bold reflect the highest loading for each variable. (DOCX) [file pone.0215534.s002.docx]

| **S2 File. Component loadings for principal component analysis (PCA) model for cardiovascular responsiveness** | | | | | |
| --- | --- | --- | --- | --- | --- |
|  | Comp. 1 | Comp. 2 | Comp. 3 | Comp. 4 | Comp. 5 |
| Mean SBP | -.007 | **.827** | .006 | -.027 | -.062 |
| Mean DBP | -.093 | **.728** | .089 | -.108 | -.097 |
| Mean MAP | -.050 | **.839** | .050 | -.069 | -.084 |
| Mean HR | -.218 | .117 | **.782** | -.316 | -.163 |
| Stroop Color-Word Mean SBP | -.015 | **.903** | .003 | -.008 | .032 |
| Stroop Color-Word Mean DBP | -.093 | **.885** | .061 | -.054 | -.015 |
| Stroop Color-Word Mean MAP | -.043 | **.954** | .018 | -.037 | .012 |
| Stroop Color-Word Mean HR | -.222 | .065 | **.891** | -.114 | -.142 |
| Stroop Pain-Affect Mean SBP | -.010 | **.899** | -.001 | .010 | .035 |
| Stroop Pain-Affect Mean DBP | -.112 | **.863** | .081 | -.055 | -.017 |
| Stroop Pain-Affect Mean MAP | -.053 | **.950** | .020 | -.031 | .011 |
| Stroop Pain-Affect Mean HR | -.243 | .056 | **.911** | -.096 | -.146 |
| Baseline Mean HR | -.212 | .094 | **.766** | -.269 | -.160 |
| Baseline SDNN | .308 | -.066 | -.182 | **.782** | .123 |
| Baseline RMSSD | .266 | -.071 | -.175 | **.584** | .055 |
| Baseline LogTP | .300 | -.057 | -.125 | **.888** | .165 |
| Baseline LogVLF | .202 | -.017 | -.206 | **.740** | .191 |
| Baseline LogLF | .275 | -.024 | -.060 | **.835** | .170 |
| Baseline LogHF | .341 | -.129 | -.041 | **.772** | .104 |
| Orthostatic Mean HR | -.104 | -.065 | **.766** | .015 | -.307 |
| Orthostatic SDNN | .302 | -.027 | -.332 | .161 | **.793** |
| Orthostatic RMSSD | .338 | .031 | -.174 | .006 | **.608** |
| Orthostatic LogTP | .287 | -.065 | -.240 | .228 | **.853** |
| Orthostatic LogVLF | .193 | -.064 | -.333 | .186 | **.783** |
| Orthostatic LogLF | .275 | -.082 | -.086 | .245 | **.756** |
| Orthostatic LogHF | .386 | .008 | -.092 | .108 | **.694** |
| Stroop Color-Word Mean HR | -.222 | .065 | **.891** | -.114 | -.142 |
| Stroop Color-Word SDNN | **.870** | -.037 | -.185 | .187 | .185 |
| Stroop Color-Word RMSSD | **.805** | -.029 | -.207 | .107 | .093 |
| Stroop Color-Word LogTP | **.828** | -.070 | -.167 | .279 | .240 |
| Stroop Color-Word LogVLF | **.605** | -.047 | -.183 | .253 | .228 |
| Stroop Color-Word LogLF | **.739** | -.056 | -.064 | .271 | .251 |
| Stroop Color-Word LogHF | **.816** | -.088 | -.185 | .214 | .152 |
| Stroop Pain-Affect Mean HR | -.243 | .056 | **.911** | -.096 | -.146 |
| Stroop Pain-Affect SDNN | **.844** | -.053 | -.188 | .164 | .193 |
| Stroop Pain-Affect RMSSD | **.838** | -.039 | -.243 | .094 | .133 |
| Stroop Pain-Affect LogTP | **.815** | -.077 | -.189 | .263 | .234 |
| Stroop Pain-Affect LogVLF | **.570** | -.048 | -.213 | .239 | .207 |
| Stroop Pain-Affect LogLF | **.725** | -.068 | -.074 | .275 | .232 |
| Stroop Pain-Affect LogHF | **.819** | -.095 | -.205 | .193 | .161 |
| Cumulative variance | .22 | .39 | .54 | .66 | .76 |
| Cronbach's alpha | .94 | .96 | .96 | .89 | .89 |
| Note. Numbers in bold reflect the highest loading for each variable. | | | |  |  |
