# Supplementary material for: Racial/ethnic differences in experimental pain sensitivity and associated factors – Cardiovascular responsiveness and psychological status
Source: PLoS One. 2019 Apr 18;14(4):e0215534. doi: 10.1371/journal.pone.0215534 (PMC6472780; doi:10.1371/journal.pone.0215534)
Supplement: S3 File — Note. Numbers in bold reflect the highest loading for each variable. (DOCX) [file pone.0215534.s003.docx]

| **S3 File. Component loadings for principal component analysis (PCA) model for psychological status** | | | |
| --- | --- | --- | --- |
|  | Comp. 1 | Comp. 2 | Comp. 3 |
| Anxiety |  |  |  |
| State (present) anxiety | **.815** | -.082 | -.024 |
| Trait (general) anxiety | **.884** | .029 | .003 |
| Depression | **.752** | .093 | .062 |
| Perceived stress scale | **.838** | .029 | .014 |
| Pain catastrophizing scale |  |  |  |
| Rumination | -.016 | **.899** | -.063 |
| Magnification | .079 | **.815** | -.005 |
| Helplessness | .088 | **.857** | -.054 |
| Pain coping questionnaire |  |  |  |
| Distraction | -.056 | .253 | **.670** |
| Distancing | .066 | -.308 | **.788** |
| Ignoring | .135 | .032 | **.713** |
| Coping self-statement | -.080 | .040 | **.776** |
| Praying | -.147 | **.534** | .315 |
| Cumulative variance | .33 | .53 | .65 |
| Cronbach's alpha | .85 | .89 | .73 |
| Note. Numbers in bold reflect the highest loading for each variable. | | | |
